# Research paper efficacy and safety of telitacicept combined with standard therapy for lupus nephritis: a single-center real-world study

**DOI:** 10.3389/fimmu.2025.1645826

**Published:** 2025-09-02

**Authors:** Xiaoxia Liu, Na Li, Pengjia Wu, Longyan Qin, Youyang Fan, Jun Liu, Aifei Zhang, Lei Yang, Ting Tian, Jiashun Zeng

**Affiliations:** Department of Rheumatology and Immunology, Affiliated Hospital of Guizhou Medical University, Guiyang, Guizhou, China

**Keywords:** lupus nephritis, telitacicept, real-world study, B-cell targeted therapy, systemic lupus erythematosus

## Abstract

**Background:**

Lupus nephritis (LN) is a severe complication of systemic lupus erythematosus with suboptimal response to standard therapy. This study evaluated the efficacy and safety of telitacicept, a novel fusion protein targeting both BLyS and APRIL, combined with standard therapy in patients with active LN.

**Methods:**

This retrospective study included 67 patients with biopsy-proven LN treated with telitacicept plus standard therapy between January 2022-July 2024. Primary endpoints included renal response rates, SRI-4, and safety at weeks 12, 24, and 52.

**Findings:**

Complete and partial renal response rates were 41.8%/26.9% at week 12 (n=67), 48.9%/34.0% at week 24 (n=47), and 56.3%/37.5% at week 52 (n=16). SRI-4 response rates increased from 55.2% at week 12 to 75.0% at week 52 (p=0.035 for trend). Proteinuria decreased significantly from 2.69g/24h at baseline to 0.51g/24h at week 24 (p<0.001). Median prednisone dose decreased from 40mg at baseline to 7.5mg at week 52 (p<0.001). Thirteen adverse events occurred with no serious events reported.

**Interpretation:**

Telitacicept combined with standard therapy demonstrated significant efficacy in LN with an acceptable safety profile, achieving high response rates, reduced proteinuria, improved immunological parameters, and substantial glucocorticoid-sparing effects.

## Introduction

Systemic lupus erythematosus (SLE) is a chronic autoimmune disease characterized by dysregulated immune responses, impaired immune tolerance, and production of pathogenic autoantibodies, resulting in multi-organ damage (1). Lupus nephritis (LN), one of the most common and severe complications of SLE, affects approximately 25-60% of SLE patients (2, 3). Despite therapeutic advances, 10-30% of patients with LN progress to end-stage kidney disease (4, 5), making LN a leading cause of mortality in SLE.

Although the pathogenesis of SLE and LN remains incompletely understood, substantial evidence indicates that aberrant B-cell activation represents a critical pathogenic mechanism in LN (6). In LN patients, hyperactivated B cells differentiate into plasma cells that produce pathogenic autoantibodies, forming immune complexes that deposit in glomerular capillary loops. These immune complexes activate the complement system and trigger inflammatory cascades, ultimately driving LN onset and progression (7).

Current standard therapy for LN primarily consists of glucocorticoids combined with immunosuppressants. However, this approach achieves complete remission in only 20-30% of patients after six months of continuous treatment (8). Additionally, prolonged high-dose glucocorticoid use frequently leads to significant adverse effects including Cushing syndrome, infections, and avascular necrosis of the femoral head. The 2024 Kidney Disease: Improving Global Outcomes (KDIGO) guidelines now recommend glucocorticoids combined with immunosuppressants and/or B cell-targeted biological agents such as belimumab for induction therapy in proliferative LN (9). The BLISS-LN trial—a multicenter, randomized, double-blind, placebo-controlled phase 3 study—demonstrated that adding belimumab to standard therapy improved primary renal response rates to 51% in Asian LN patients at week 104 (2). These findings highlight the potential benefit of incorporating biological agents into LN treatment regimens.

Telitacicept, a novel recombinant TACI-Fc fusion protein, represents an innovative approach to B cell-targeted therapy for autoimmune diseases. Unlike belimumab which targets only B lymphocyte stimulator (BLyS), telitacicept simultaneously inhibits both BLyS and a proliferation-inducing ligand (APRIL), thereby blocking B cell maturation, plasma cell generation, and subsequent autoantibody production through dual-target mechanism. Phase II and III clinical trials of telitacicept in SLE have demonstrated promising SRI-4 response rates of 68.3% and 82.6%, respectively (10, 11). However, robust real-world evidence for telitacicept in LN treatment remains limited.

This retrospective study aimed to comprehensively evaluate the efficacy and safety of telitacicept combined with standard therapy in patients with active LN in a real-world clinical setting. We assessed renal responses, overall disease activity, immunological parameters, glucocorticoid-sparing effects, and treatment-related adverse events to provide insights into the potential role of telitacicept in LN management.

## Materials and methods

### Study design and participants

This single-center retrospective study analyzed data from patients with active lupus nephritis (LN) who received telitacicept combined with standard therapy at the Department of Rheumatology and Immunology, Affiliated Hospital of Guizhou Medical University, between January 2022 and July 2024. This timeframe represents the period since telitacicept was approved and became available for clinical use at our institution. The study protocol was approved by the Ethics Committee of the Affiliated Hospital of Guizhou Medical University [2024142K].

### Inclusion and exclusion criteria

Patients were included if they: (1) were ≥18 years of age, regardless of sex; (2) fulfilled the 2019 European League Against Rheumatism (EULAR)/American College of Rheumatology (ACR) classification criteria for SLE; (3) had kidney involvement confirmed by renal biopsy with 24-hour urinary protein ≥0.5 g or protein/creatinine ratio (PCR) ≥0.5 g/g; and (4) received telitacicept treatment for ≥12 weeks.

Patients were excluded if they had: (1) primary kidney disease or other secondary glomerular diseases (e.g., diabetic kidney disease, hypertensive nephropathy, Henoch-Schönlein purpura nephritis); (2) concomitant rheumatic or autoimmune disorders (e.g., dermatomyositis, rheumatoid arthritis, systemic vasculitis); (3) uncontrolled hypertension (blood pressure >140/90 mmHg despite antihypertensive therapy), uncontrolled diabetes (HbA1c >8%), significant hematological disorders (hemoglobin <80 g/L, white blood cell count <3.0×10^9/L, or platelet count <50×10^9/L), or severe cardiopulmonary dysfunction (NYHA class III-IV); (4) abnormal renal function before telitacicept initiation (serum creatinine >265 μmol/L or estimated glomerular filtration rate [eGFR] <30 mL/min/1.73 m²) or ongoing renal replacement therapy; (5) history of major organ transplantation or hematopoietic stem cell/bone marrow transplantation; or (6) pregnancy or lactation.

### Treatment protocol

Telitacicept was administered subcutaneously at doses of 160 mg or 80 mg once weekly for at least 12 weeks. The initial dose was determined primarily based on disease severity, body weight, and the treating physician’s clinical judgment. The 160 mg dose was generally preferred for patients with high disease activity (SLEDAI-2K ≥10) or severe proteinuria (>3.5 g/24h), while the 80 mg dose was typically used for patients with moderate disease activity or lower body weight (<50 kg). Dosage adjustments were made according to patients’ clinical response, laboratory parameters, and adverse events at the discretion of the treating physicians.

Standard therapy consisted of glucocorticoids combined with immunosuppressants. Initial prednisone or equivalent doses ranged from 0.5-1.0 mg/kg/day, with subsequent tapering based on clinical response. Immunosuppressive agents included cyclophosphamide (CTX, 0.4-0.6 g/m² intravenously every 2–4 weeks), mycophenolate mofetil (MMF, 1.0-2.0 g/day orally in divided doses), tacrolimus (TAC, 0.05-0.1 mg/kg/day, targeting trough levels of 4–7 ng/mL), cyclosporine A (CsA, 3–5 mg/kg/day, targeting trough levels of 100–150 ng/mL), or combinations thereof. Hydroxychloroquine (≤5 mg/kg/day) was administered to all patients unless contraindicated.

### Efficacy assessments

Treatment efficacy for SLE was evaluated using the SLE Disease Activity Index 2000 (SLEDAI-2K) score and SRI-4 response rate. SRI-4 response was defined as: (1) ≥4-point reduction in SLEDAI-2K score compared to baseline; (2) no new British Isles Lupus Assessment Group (BILAG) A organ domain score or no more than one new BILAG B organ domain score compared to baseline; and (3) no worsening in Physician’s Global Assessment (PGA) score (increase <0.3 from baseline).

Renal response was categorized according to international consensus definitions (12) as follows: (1) Complete renal response (CR): 24-hour urinary protein <0.5 g or PCR <0.5 g/g, with normal or stable renal function (eGFR within ±10-15% of baseline or stable); (2) Partial renal response (PR): ≥50% reduction in 24-hour urinary protein to <3.0 g or ≥50% reduction in PCR to <3.0 g/g, with normal or stable renal function (eGFR within ±10-15% of baseline or stable); (3) No renal response (NR): failure to meet criteria for partial or complete renal response. Overall response rate was calculated as (number of patients with CR + number of patients with PR)/total number of patients × 100%.

### Data collection

We collected demographic and clinical data including sex, age, LN disease duration, renal pathology classification, extra-renal organ involvement, glucocorticoid dosage, immunosuppressant regimens, and SLEDAI-2K scores. Laboratory parameters were assessed at baseline and at weeks 12, 24, and 52, including 24-hour urinary protein, serum albumin, lymphocyte count, hemoglobin, serum creatinine, eGFR, anti-dsDNA antibodies, and complement C3 levels. Renal response rates were also evaluated at these time points.

### Safety assessment

Safety evaluations included assessment of treatment-related adverse events such as infusion/injection reactions, hypersensitivity reactions, infections, cytopenias (leukopenia, anemia, thrombocytopenia), liver enzyme elevations, and gastrointestinal symptoms. Adverse events were graded according to the Common Terminology Criteria for Adverse Events (CTCAE) version 5.0. Serious adverse events were defined as events resulting in death, life-threatening conditions, hospitalization or prolongation of existing hospitalization, persistent or significant disability, or congenital anomaly/birth defect.

### Statistical analysis

Statistical analyses were performed using SPSS version 26.0 (IBM Corp., Armonk, NY, USA). No formal sample size calculation was performed for this retrospective study; all eligible patients during the study period were included in the analysis. For longitudinal comparisons at different time points, baseline values were recalculated using only patients who completed the respective follow-up period (67 patients at 12 weeks, 47 patients at 24 weeks, and 16 patients at 52 weeks) to ensure appropriate paired statistical analyses and avoid bias from missing data. Normally or approximately normally distributed continuous variables were expressed as mean ± standard deviation and compared using paired t-tests for within-group comparisons or independent t-tests for between-group comparisons. Non-normally distributed continuous variables were presented as median (interquartile range [IQR]) and compared using Wilcoxon signed-rank tests for within-group comparisons or Mann-Whitney U tests for between-group comparisons. Categorical variables were expressed as counts and percentages and compared using chi-square tests or Fisher’s exact test as appropriate. Missing data were not imputed; analyses were conducted on available data. A two-sided p-value ≤0.05 was considered statistically significant.

## Results

### Patient characteristics

We initially screened 101 patients, of whom 34 were excluded (2 receiving hemodialysis, 14 without renal biopsies, 14 with treatment duration <12 weeks, and 6 with incomplete follow-up data). The final analysis included 67 patients, 61 (91%) of whom were female. The mean age at telitacicept initiation was 34.13 ± 10.25 years, with a median disease duration of 17 months (IQR 6-40).

All 67 patients underwent renal biopsy. The predominant pathological classifications were class IV (n=38, 56.7%), followed by class IV+V (n=10, 14.9%), class III (n=8, 11.9%), class V (n=7, 10.4%), and class III+V (n=4, 6.0%). Extra-renal manifestations included mucocutaneous involvement (n=26, 38.8%), musculoskeletal involvement (n=38, 56.7%), hematological abnormalities (n=31, 46.3%), neuropsychiatric symptoms (n=2, 3.0%), and serositis (n=8, 11.9%).

All patients received glucocorticoids, with a median baseline prednisone dose of 40 mg (IQR 17.5-50). Concurrent medications included hydroxychloroquine in all patients and various immunosuppressive regimens: cyclophosphamide (n=7, 10.4%), mycophenolate mofetil (n=34, 50.7%), tacrolimus or cyclosporine (n=6, 9.0%), mycophenolate mofetil plus tacrolimus (n=15, 22.4%), and hydroxychloroquine alone (n=5, 7.5%). The mean baseline SLEDAI-2K score was 12.04 ± 3.34, with all 67 patients having moderate-to-severe disease activity (SLEDAI-2K ≥6) and 38 (56.7%) having severe disease activity (SLEDAI-2K ≥12). Of the 67 enrolled patients, 47 completed 24 weeks of treatment, while 16 completed 52 weeks of treatment (**Table 1**).

**Table 1:** Baseline demographic and clinical characteristics of patients with lupus nephritis treated with telitacicept (N=67).

Characteristic	Value
Female sex, n (%)	61 (91.0)
Age at telitacicept initiation, years	34.13 ± 10.25
Disease duration, months	17 (6-40)
Renal histopathology class, n (%)	
Class III	8 (11.9)
Class IV	38 (56.7)
Class V	7 (10.4)
Class III+V	4 (6.0)
Class IV+V	10 (14.9)
Extra-renal manifestations, n (%)	
Mucocutaneous	26 (38.3)
Musculoskeletal	38 (56.7)
Haematological	31 (46.3)
Neuropsychiatric	2 (3.0)
Serositis	8 (11.9)
Prednisone dose, mg/day	40 (17.5-50)
Background medications, n (%)	
Hydroxychloroquine	67 (100)
Cyclophosphamide	7 (10.4)
Mycophenolate mofetil	34 (50.7)
Tacrolimus or cyclosporine	6 (9.0)
Mycophenolate mofetil + tacrolimus	15 (22.4)
SLEDAI-2K score	12.04 ± 3.34
24-hour urinary protein, g	2.69 (1.25-4.43)
Complement C3, g/L	0.61 (0.43-0.87)
Complement C4, g/L	0.15 (0.08-0.19)
Immunoglobulin G, g/L	10.1 (6.98-13.1)
Immunoglobulin A, g/L	2.17 (1.73-2.83)
Immunoglobulin M, g/L	0.93 (0.57-1.28)

Data are presented as n (%), mean ± SD, or median (IQR). SLEDAI-2K, Systemic Lupus Erythematosus Disease Activity Index 2000.

Due to progressive patient attrition during follow-up (from 67 patients at 12 weeks to 47 patients at 24 weeks and 16 patients at 52 weeks), all longitudinal analyses were performed using paired comparisons with baseline values recalculated for each time point based on patients with available data at that specific time point.

### Renal response

At weeks 12, 24, and 52, complete renal response (CR) rates were 41.8% (28/67), 48.9% (23/47), and 56.3% (9/16), respectively; partial renal response (PR) rates were 26.9% (18/67), 34.0% (16/47), and 37.5% (6/16), respectively; and no response (NR) rates were 31.3% (21/67), 17.0% (8/47), and 6.2% (1/16), respectively. The overall response rates (CR+PR) increased over time: 68.7%, 83.0%, and 93.8% at weeks 12, 24, and 52, respectively.

**Figure 1:**
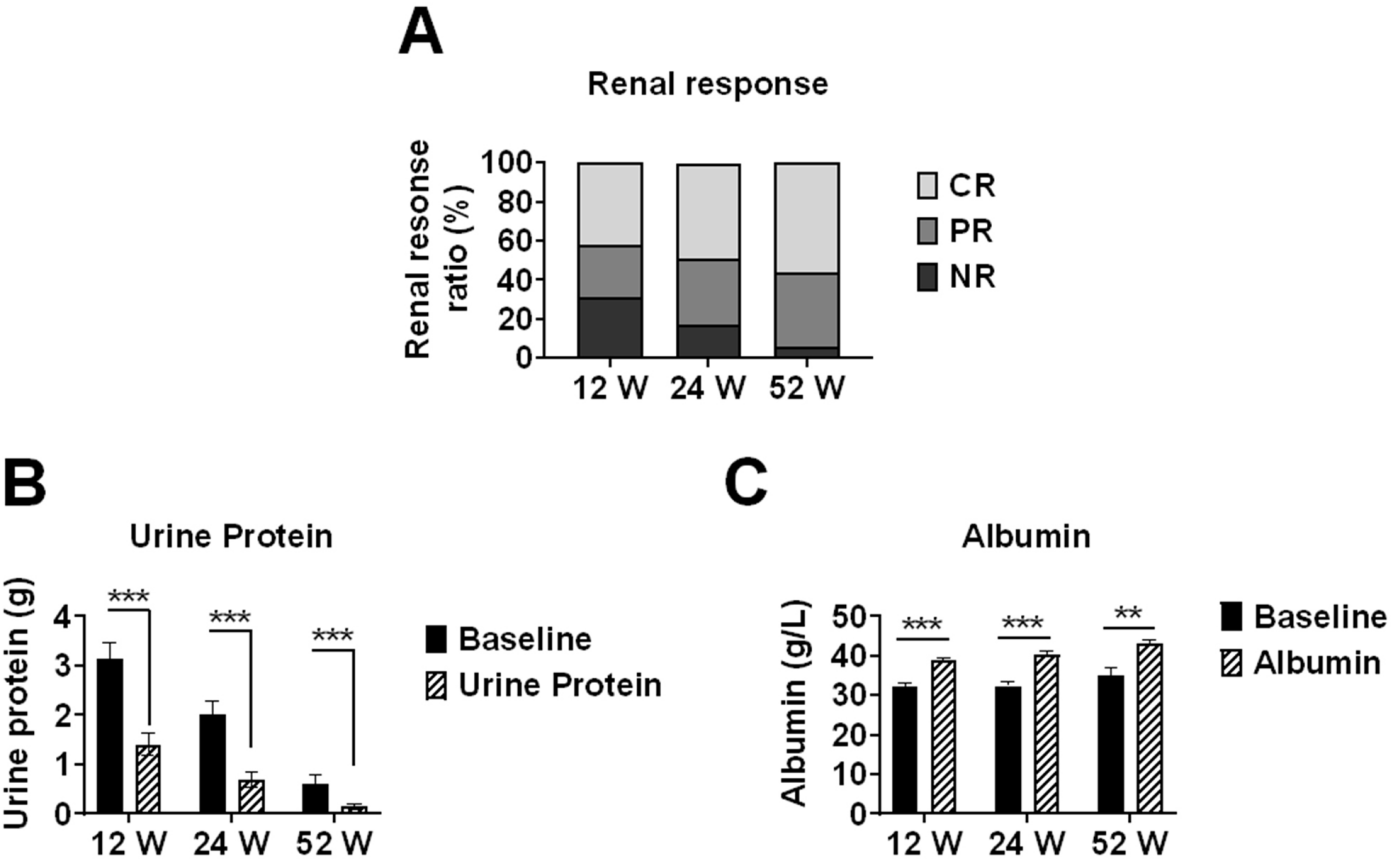
Renal response rates and changes in proteinuria and serum albumin levels in lupus nephritis patients treated with telitacicept. Renal outcomes in lupus nephritis patients receiving telitacicept treatment at baseline, 12, 24, and 52 weeks. **(A)** Renal response rates, including complete response (CR), partial response (PR), and non-response (NR). **(B)** 24-hour urinary protein levels. **(C)** Serum albumin levels. Baseline values in **(B, C)** were recalculated for each time point using only patients who completed the respective follow-up period (n=67 at 12 weeks, n=47 at 24 weeks, n=16 at 52 weeks) to ensure accurate paired comparisons. Data are presented as medians with interquartile ranges for continuous variables. **p<0.01, ***p<0.001 compared with baseline values.

Median 24-hour urinary protein decreased significantly from baseline (2.69 g) to 0.76 g (IQR 0.29-1.63) at week 12 (n=67), 0.51 g (IQR 0.27-1.11) at week 24 (n=47), and 0.29 g (IQR 0.10-0.99) at week 52 (n=16) (all p<0.001 *vs* respective baseline). Correspondingly, median serum albumin increased significantly from respective baseline values to 39.6 g/L (IQR 35.57-42.88) at week 12 (n=67), 41.8 g/L (IQR 38.3-44.0) at week 24 (n=47), and 43.9 g/L (IQR 39.25-46.05) at week 52 (n=16) (all p<0.001 *vs* respective baseline). Correspondingly, median serum albumin increased significantly from baseline to 39.6 g/L (IQR 35.57-42.88) at week 12, 41.8 g/L (IQR 38.3-44.0) at week 24, and 43.9 g/L (IQR 39.25-46.05) at week 52 (all p<0.001 *vs* baseline) (**Figure 1**).

### Overall treatment response

**Figure 2:**
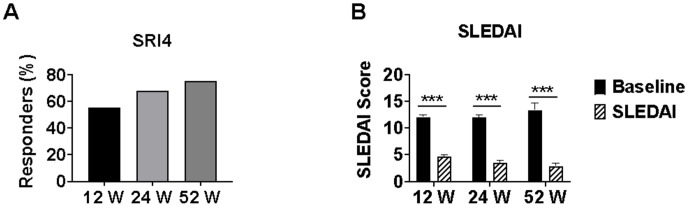
SLE disease activity measures in lupus nephritis patients treated with telitacicept. Changes in SLE disease activity during telitacicept treatment at baseline, 12, 24, and 52 weeks. **(A)** SRI-4 response rates. **(B)** SLEDAI-2K scores. Baseline values in panel B were recalculated for each time point using only patients who completed the respective follow-up period (n=67 at 12 weeks, n=47 at 24 weeks, n=16 at 52 weeks) to ensure accurate paired comparisons. Data are presented as mean ± SD for SLEDAI-2K scores. ***p<0.001 compared with baseline values. SRI-4, SLE Responder Index 4. SLEDAI-2K, Systemic Lupus Erythematosus Disease Activity Index 2000.

SRI-4 response rates were 55.2% (37/67) at week 12 and increased to 68.1% (32/47) at week 24, and 75.0% (12/16) at week 52 (p=0.035 for trend). Mean SLEDAI-2K scores decreased significantly from baseline (12.04 ± 3.34) to 4.7 ± 2.84 at week 12 (n=67), 3.51 ± 2.56 at week 24 (n=47), and 2.88 ± 2.19 at week 52 (n=16) (all p<0.001 *vs* respective baseline), representing mean reductions of 60.9%, 70.8%, and 76.1%, respectively. This improvement in SLEDAI-2K scores indicates a substantial reduction in overall disease activity with continued telitacicept treatment (**Figure 2**).

### Immunological parameters

In patients with available data, anti-dsDNA antibody negativity rates increased from baseline to 20.9% (14/67) at week 12, 25.53% (12/47) at week 24, and 31.25% (5/16) at week 52. Median complement C3 levels increased significantly from respective baseline values to 0.88 g/L (IQR 0.74-1.03) at week 12 (n=67), 0.96 g/L (IQR 0.82-1.04) at week 24 (n=47), and 0.85 g/L (IQR 0.79-1.01) at week 52 (n=16) (all p<0.05 *vs* respective baseline).

**Figure 3:**
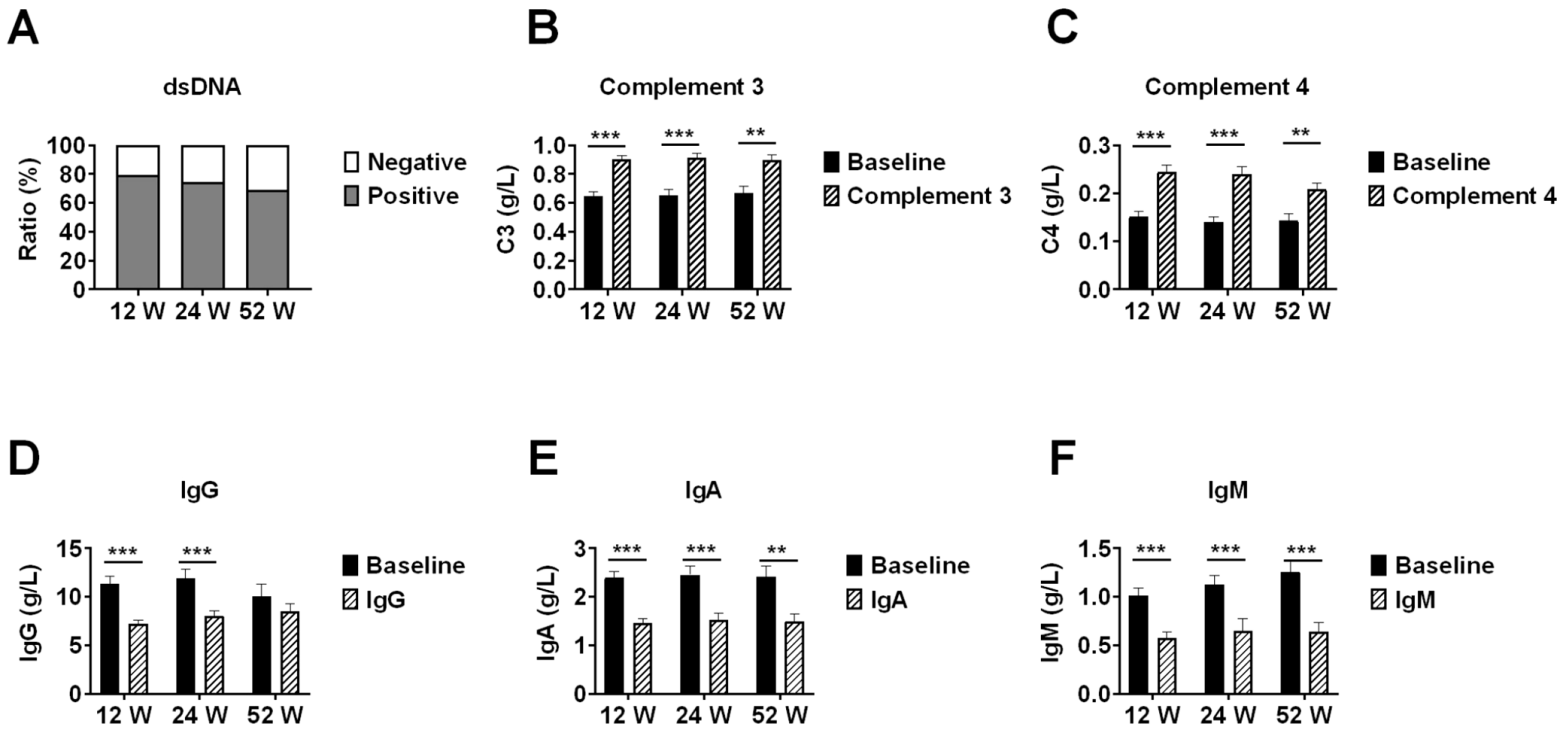
Immunological parameter changes in lupus nephritis patients during telitacicept treatment. Changes in immunological parameters during telitacicept treatment at baseline, 12, 24, and 52 weeks. **(A)** Anti-dsDNA antibody negativity rates. **(B)** Complement C3 levels. **(C)** Complement C4 levels. **(D)** Immunoglobulin G levels. **(E)** Immunoglobulin A levels. **(F)** Immunoglobulin M levels. Baseline values in panels **(B-F)** were recalculated for each time point using only patients who completed the respective follow-up period (n=67 at 12 weeks, n=47 at 24 weeks, n=16 at 52 weeks) to ensure accurate paired comparisons. Data are presented as medians with interquartile ranges. **p<0.01, ***p<0.001 compared with baseline values; ns, not significant.

Similarly, complement C4 levels increased from baseline to 0.23 g/L (IQR 0.18-0.26) at week 12, 0.23 g/L (IQR 0.19-0.27) at week 24, and 0.22 g/L (IQR 0.18-0.25) at week 52. Immunoglobulin levels showed significant changes: IgG decreased from baseline to 6.51 g/L (IQR 5.16-8.64) at week 12 (p<0.001), 7.19 g/L (IQR 6.01-9.08) at week 24 (p<0.001), and 8.06 g/L (IQR 6.60-10.68) at week 52 (p=0.179); IgA decreased from baseline to 1.38 g/L (IQR 0.91-1.90) at week 12, 1.36 g/L (IQR 0.99-1.86) at week 24, and 1.43 g/L (IQR 0.95-1.99) at week 52 (all p<0.05); and IgM decreased from baseline to 0.42 g/L (IQR 0.27-0.80) at week 12, 0.46 g/L (IQR 0.31-0.74) at week 24, and 0.62 g/L (IQR 0.28-0.93) at week 52 (all p<0.001) (**Figure 3**).

### Response by histological class

Among the 47 patients who completed 24 weeks of treatment, class IV LN patients (n=26) showed CR and PR rates of 53.8% (14/26) and 34.6% (9/26), respectively, resulting in an overall response rate of 88.4%. In class IV+V LN (n=7), one patient achieved CR (14.3%) and five achieved PR (71.4%), with an overall response rate of 85.7%. In class III LN (n=6), five patients achieved CR (83.3%) while one had NR, demonstrating the highest CR rate among all pathological classes. In class V LN (n=5), one patient achieved CR (20.0%), two achieved PR (40.0%), and two had NR (40.0%), representing the lowest overall response rate (60.0%) among the pathological classes (**Table 2**).

**Table 2:** Renal responses by histological class at week 24 in patients treated with telitacicept (N=47).

Histological class (n)	Complete response, n (%)	Partial response, n (%)	No response, n (%)	24-hour urinary protein, g*
Class III (n=6)	5 (83.3)	0	1 (16.7)	1.17 ± 2.32
Class IV (n=26)	14 (53.8)	9 (34.6)	3 (11.5)	0.61 ± 0.49
Class V (n=5)	1 (20.0)	2 (40.0)	2 (40.0)	1.05 ± 0.91
Class III+V (n=3)	2 (66.7)	0	1 (33.3)	1.73 ± 2.68
Class IV+V (n=7)	1 (14.3)	5 (71.4)	1 (14.3)	1.88 ± 2.44

*Data presented as mean ± SD.

### Subgroup analysis by immunosuppressive regimen

At week 12, patients were divided into three groups based on their immunosuppressive regimens: prednisone plus cyclophosphamide (CTX group, n=6), prednisone plus mycophenolate mofetil (MMF group, n=35), and prednisone plus mycophenolate mofetil and tacrolimus (MMF+CNI group, n=15). At baseline, these groups were comparable in age, sex, disease duration, SLEDAI-2K scores, proteinuria, immunoglobulin levels, complement levels, and prednisone dosage, except that the MMF+CNI group had significantly lower serum albumin compared to the MMF group.

At week 12, the median 24-hour urinary protein was significantly lower in the MMF group (0.38 g [IQR 0.18-1.48]) compared to both the CTX group (2.16 g [IQR 0.43-3.49]) and the MMF+CNI group (1.45 g [IQR 0.41-2.57]) (both p<0.05). Similarly, the median SLEDAI-2K score was significantly lower in the MMF group (4.0 [IQR 2.0-6.0]) compared to the MMF+CNI group (6.0 [IQR 4.0-8.0]) (p<0.05).

**Figure 4:**
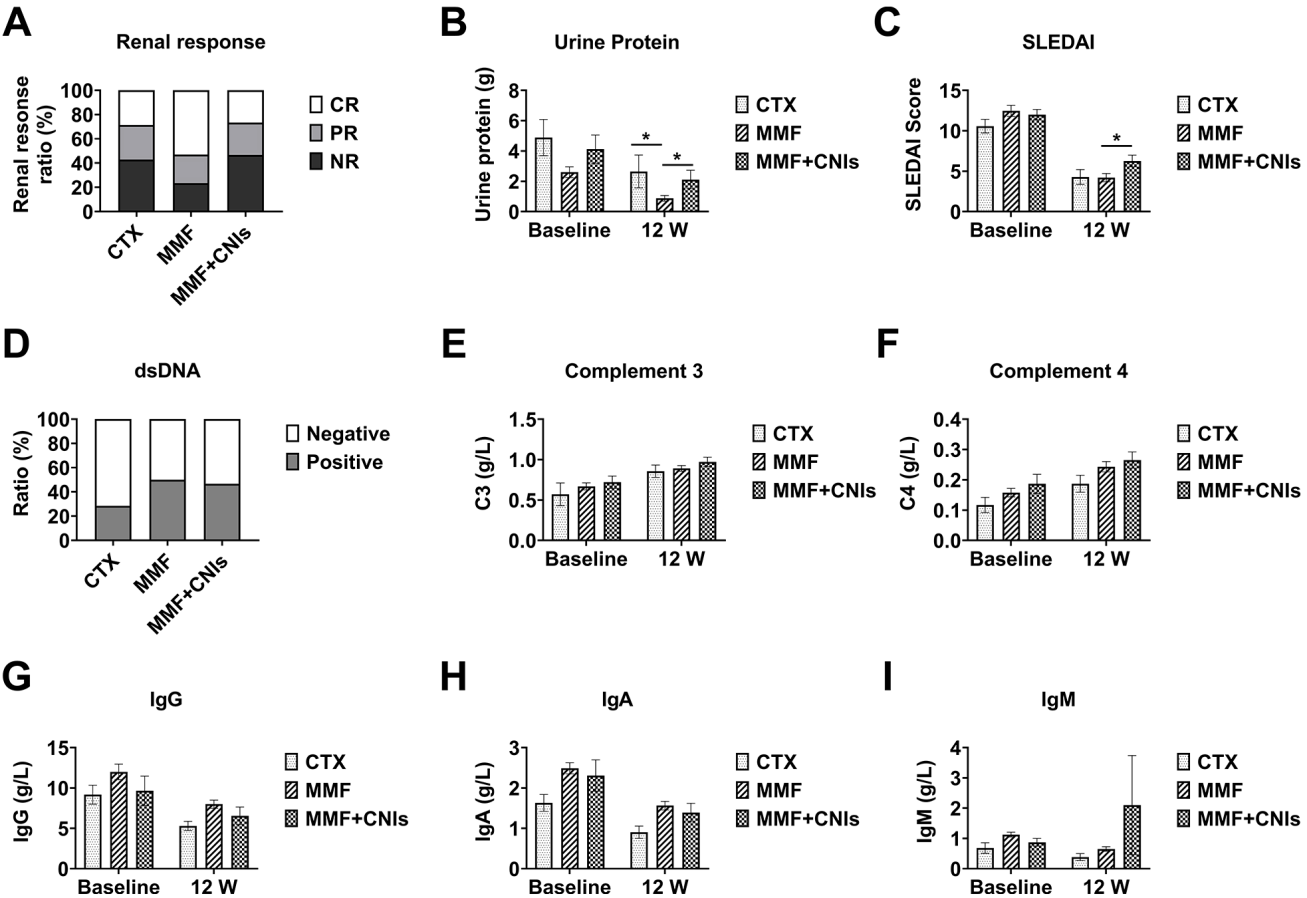
Efficacy of different immunosuppressive regimens combined with telitacicept in lupus nephritis patients at 12 weeks. Clinical and immunological outcomes at week 12 in lupus nephritis patients receiving telitacicept combined with different immunosuppressive regimens: corticosteroids plus cyclophosphamide (CTX, n=6), corticosteroids plus mycophenolate mofetil (MMF, n=35), and corticosteroids plus mycophenolate mofetil and tacrolimus (MMF+CNI, n=15). **(A)** Renal response rates. **(B)** 24-hour urinary protein excretion. **(C)** SLEDAI scores. **(D)** Anti-dsDNA antibody levels. **(E)** Complement C3 levels. **(F)** Complement C4 levels. **(G)** Immunoglobulin G levels. **(H)** Immunoglobulin A levels. **(I)** Immunoglobulin M levels. Data are presented as medians with interquartile ranges for continuous variables. *p<0.05 for between-group comparisons as indicated.

**Figure 5:**
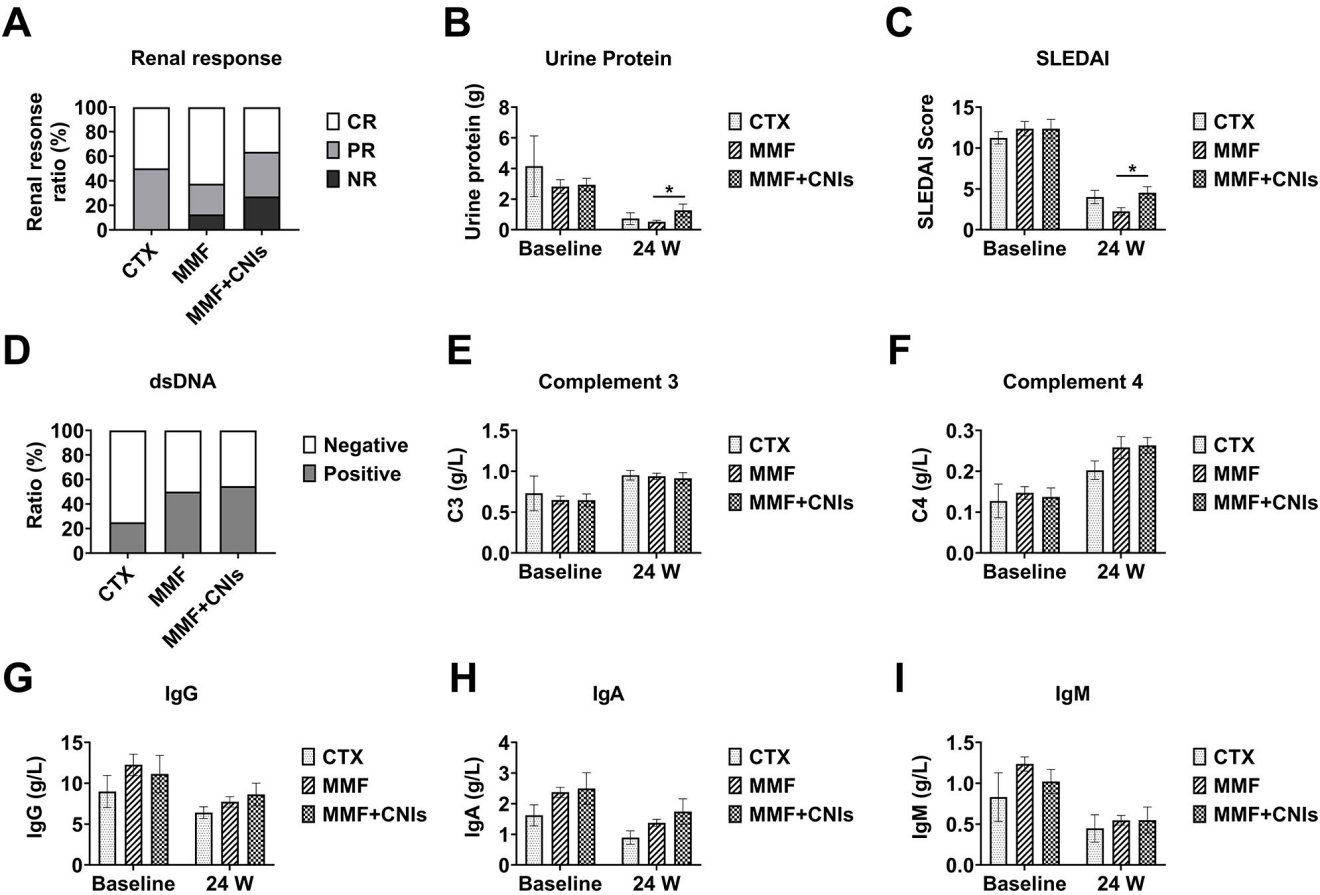
Efficacy of different immunosuppressive regimens combined with telitacicept in lupus nephritis patients at 24 weeks. Clinical and immunological outcomes at week 24 in lupus nephritis patients receiving telitacicept combined with different immunosuppressive regimens: corticosteroids plus cyclophosphamide (CTX, n=4), corticosteroids plus mycophenolate mofetil (MMF, n=24), and corticosteroids plus mycophenolate mofetil and tacrolimus (MMF+CNI, n=11). **(A)** Renal response rates. **(B)** 24-hour urinary protein excretion. **(C)** SLEDAI scores. **(D)** Anti-dsDNA antibody levels. **(E)** Complement C3 levels. **(F)** Complement C4 levels. **(G)** Immunoglobulin G levels. **(H)** Immunoglobulin A levels. **(I)** Immunoglobulin M levels. Data are presented as medians with interquartile ranges for continuous variables. *p<0.05 for between-group comparisons as indicated.

At week 24, the CTX group (n=4), MMF group (n=24), and MMF+CNI group (n=11) showed median 24-hour urinary protein of 0.497 g (IQR 0.16-1.53), 0.40 g (IQR 0.16-0.63), and 0.89 g (IQR 0.34-1.36), respectively, with the MMF group showing significantly lower values than the MMF+CNI group (p<0.05). The median SLEDAI-2K scores were 4.0 (IQR 2.5-5.5), 2.0 (IQR 0.0-4.0), and 4.0 (IQR 4.0-6.0), respectively, with the MMF group again showing significantly lower scores than the MMF+CNI group (p<0.05). No significant differences in adverse events were observed between the three groups (**Figures 4**, **5**).

### Glucocorticoid dose reduction and changes in immunosuppressive regimens

The median prednisone dose decreased significantly from baseline (40 mg [IQR 17.5-50]) to 15 mg (IQR 10-25) at week 12, 10 mg (IQR 7.5-15) at week 24, and 7.5 mg (IQR 5.0-9.375) at week 52 (all p<0.001 *vs* baseline). This represents a median reduction of 62.5%, 75.0%, and 81.3% from baseline at weeks 12, 24, and 52, respectively, demonstrating a substantial glucocorticoid-sparing effect with telitacicept treatment.

**Figure 6:**
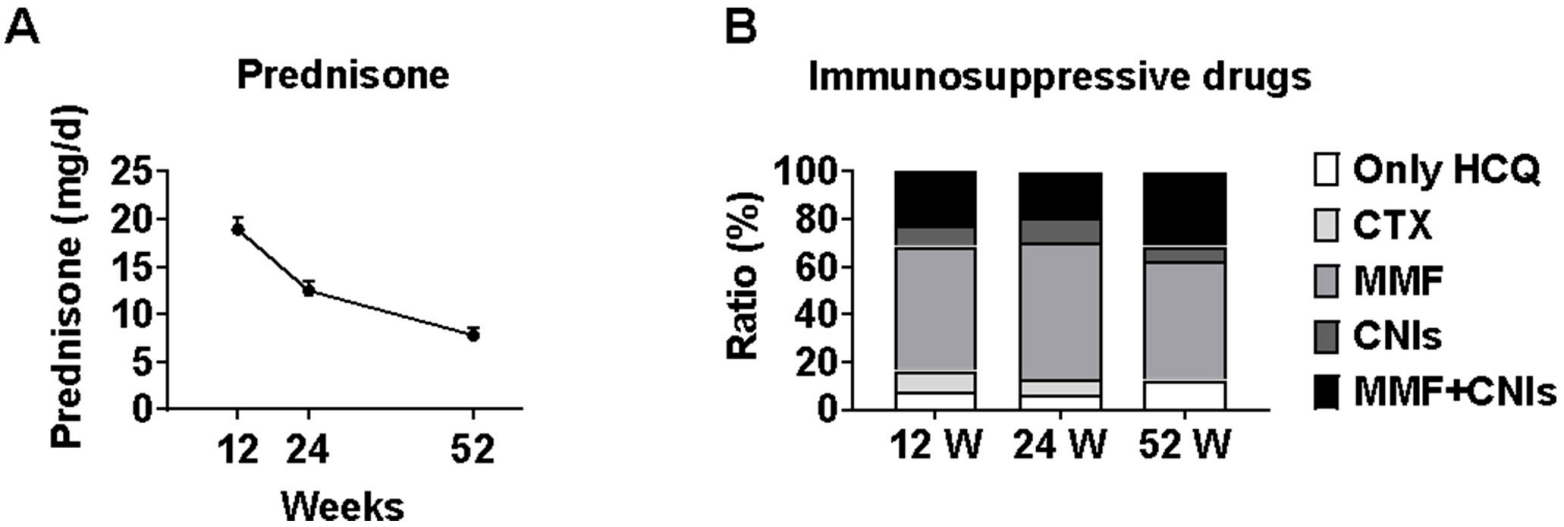
Changes in glucocorticoid dosage and immunosuppressive regimens during telitacicept treatment. Prednisone dose reduction and immunosuppressive regimen changes during telitacicept treatment. **(A)** Prednisone dosage at baseline, 12, 24, and 52 weeks. Data are presented as medians with interquartile ranges. **(B)** Changes in immunosuppressive regimens throughout the study period, showing transitions between different therapies.

Changes in immunosuppressive regimens were minimal: by week 12, one patient switched from cyclophosphamide to mycophenolate mofetil due to gastrointestinal reactions; by week 24, one patient switched from mycophenolate mofetil plus tacrolimus to tacrolimus alone, another from mycophenolate mofetil plus tacrolimus to mycophenolate mofetil alone, and one from cyclophosphamide to mycophenolate mofetil; by week 52, one patient discontinued mycophenolate mofetil, and another switched from mycophenolate mofetil to mycophenolate mofetil plus tacrolimus (**Figure 6**).

### Safety

Thirteen adverse events were reported during the follow-up period: upper respiratory tract infections (n=3), pneumonia (n=3), urinary tract infections (n=3), injection site reactions (n=1), and herpes zoster (n=3). All events resolved with appropriate treatment, and no serious adverse events were reported.

## Discussion

Lupus nephritis (LN) remains one of the most challenging manifestations of systemic lupus erythematosus (SLE), with substantial morbidity and mortality despite standard therapeutic approaches. Even with aggressive treatment using glucocorticoids and immunosuppressants, approximately 60% of patients fail to achieve complete renal response (13–15), and those without remission have a 10-year renal survival rate of only 19% (16). Moreover, LN is characterized by high relapse rates (27-66%) (2, 17), which further contribute to organ damage and poor outcomes. B cells play a central role in LN pathogenesis through multiple mechanisms, including loss of immune tolerance, abnormal B cell signaling, increased self-reactive B cell production, abnormal B cell apoptosis, and production of pathogenic autoantibodies that cause kidney inflammation and damage (18). Therefore, novel therapeutic agents targeting B cells offer promising approaches to reduce renal inflammation, prevent disease recurrence, and preserve long-term kidney function.

Over the past decade, advances in understanding the pathogenesis of LN have led to the development of various biologic agents targeting B cells, including inhibitors of BLyS and APRIL (19). The survival, maturation, and differentiation of B cells are regulated by these B cell-related cytokines, with increased expression of BLyS and APRIL being closely associated with LN pathogenesis (18). Telitacicept represents a novel approach to B cell-targeted therapy as a dual-target recombinant human TACI-Fc fusion protein that simultaneously inhibits both BLyS and APRIL, thereby blocking B cell maturation and plasma cell differentiation, ultimately reducing pathogenic autoantibody production and disease progression (20). This dual-targeting mechanism potentially offers advantages over single-target therapies such as belimumab, which inhibits only BLyS. Subgroup analyses from phase II and III clinical trials of telitacicept in SLE have suggested efficacy in LN patients, but comprehensive real-world evidence has been limited (10, 11).

In this real-world study evaluating telitacicept combined with standard therapy for active LN, we observed promising efficacy and safety profiles. Our findings demonstrate progressive improvement in both complete and partial renal response rates over time, with the overall response rate reaching an impressive 93.8% at 52 weeks. The significant reduction in proteinuria (median reduction of 89.2% at week 52) and improvement in serum albumin levels indicate substantial resolution of nephrotic syndrome, a key determinant of long-term renal outcomes in LN.

Our results compare favorably with traditional treatment regimens. Conventional therapy with mycophenolate mofetil typically achieves complete renal response rates of 21.6%-31.6%, while multitarget therapy with glucocorticoids combined with mycophenolate mofetil and tacrolimus yields response rates of approximately 32.6%-53.2% (21, 22). Our observed complete renal response rate of 48.9% at 24 weeks and 56.3% at 52 weeks suggests that telitacicept combined with standard therapy may offer superior efficacy, particularly in achieving complete renal remission, which is associated with better long-term outcomes. However, randomized controlled trials are needed to directly compare telitacicept with traditional treatment regimens.

Interestingly, Huang et al. reported a complete renal response rate of 73.3% in 30 LN patients treated with telitacicept for at least 24 weeks (23), which is higher than our observations. This discrepancy may be attributed to differences in patient populations, as our cohort included seven patients with refractory LN and had a predominance of class IV LN cases (56.7%), which typically show more severe kidney involvement and potentially slower treatment response.

With regard to overall SLE disease activity, our study showed progressive improvement in SRI-4 response rates (reaching 75.0% at week 52) and significant decreases in SLEDAI-2K scores. These findings align with previous reports (24, 25), including a study by Hong Zhu et al. documenting a reduction in SLEDAI scores from 13 at baseline to 4 at study endpoint in 13 LN patients treated with telitacicept (24).

In the phase III trial evaluating belimumab for LN in East Asian populations (26), the SRI-4 response rate at 52 weeks was 53.8%, whereas our study demonstrated a higher SRI-4 response rate of 75.0%. While this suggests that telitacicept may have comparable or potentially superior efficacy compared to belimumab, it is important to note that differences in study design, patient populations, and concurrent therapies limit direct comparisons. Head-to-head trials with larger sample sizes are needed to definitively establish the relative efficacy of these biological agents in SLE and LN.

Our study also revealed that telitacicept treatment at weeks 12, 24, and 52 was associated with significant increases in complement C3 and C4 levels compared to baseline, while IgG, IgA, and IgM levels decreased. It is noteworthy that at week 52, although IgG levels were lower compared to baseline, the difference was not statistically significant, possibly related to the inclusion of nine refractory LN patients among the 16 who completed 52 weeks of treatment. Of particular interest, we observed that three patients had IgG levels fluctuating between 3–4 g/L and IgM levels below 0.3 g/L after 12 weeks of telitacicept treatment, yet none experienced serious adverse events during follow-up. This raises an important clinical question: at what threshold values of immunoglobulins should clinicians intervene or adjust treatment strategies for SLE patients receiving biological agents? Currently, there is limited literature addressing this issue. Furst et al. demonstrated that infection risk, particularly fatal infections, significantly increases when IgG ≤100 mg/dL or IgM ≤20 mg/dL, while IgA levels do not correlate with infection risk (27). Therefore, defining critical threshold values for IgG and IgM to predict infection risk in SLE patients receiving immunosuppressants and/or biological agents is crucial. Additionally, we observed anti-dsDNA antibody negativity rates of 20.9%, 25.53%, and 31.25% at weeks 12, 24, and 52, respectively, consistent with previous reports (28). Since positive anti-dsDNA antibodies and persistent hypocomplementemia are important indicators for overall SLE disease activity assessment (29) and may predict disease activity and relapse, our findings suggest that telitacicept improves hypocomplementemia and reduces pathogenic antibody titers in LN patients, helping to control disease and reduce relapses.

According to the 2024 KDIGO guidelines, renal biopsy is recommended for LN patients with significant proteinuria to determine pathological type and disease severity (9). In our cohort, class IV LN was predominant (56.7%), consistent with typical LN populations. Subgroup analysis revealed differential efficacy based on histological class, with class III LN patients showing the highest complete renal response rate (83.3%) at 24 weeks, while class IV LN patients had an impressive overall response rate of 88.4%. These findings align with previous observations from the BLISS-LN trial, where Rovin et al. (30) found that belimumab showed better efficacy in proliferative LN compared to pure class V LN.

The question of which LN patients might benefit most from telitacicept remains to be fully answered. Our findings suggest particularly strong efficacy in proliferative LN, especially class III, but the small sample size of certain histological subgroups limits definitive conclusions. Larger studies specifically designed to assess the influence of renal pathological types on telitacicept treatment response are needed to optimize patient selection for this therapy.

Our subgroup analysis based on immunosuppressive regimens yielded several interesting observations. The MMF group consistently showed lower proteinuria and SLEDAI-2K scores at weeks 12 and 24 compared to the MMF+CNI group, suggesting a particularly effective synergy between telitacicept and mycophenolate mofetil. However, this finding should be interpreted considering that seven patients in the MMF+CNI group had refractory LN, potentially explaining their more modest treatment response.

It is noteworthy that the MMF+CNI group achieved an overall renal response rate of 72.7% at 24 weeks despite the inclusion of refractory cases, suggesting that the combination of telitacicept with multitarget therapy might be a viable option for difficult-to-treat LN. The absence of increased adverse events in this group is reassuring regarding the safety of combining telitacicept with intensive immunosuppression. These findings suggest that immunosuppressant selection should be tailored to disease severity and pathological type, with the potential to escalate to multitarget therapy plus telitacicept in refractory cases. Larger studies are needed to establish optimal dosing strategies and long-term outcomes of various combination approaches.

A significant finding in our study was the substantial glucocorticoid-sparing effect of telitacicept, with median prednisone doses decreasing from 40 mg at baseline to 7.5 mg at week 52. While this does not quite reach the KDIGO-recommended target of <5 mg/day (9), it represents a significant reduction compared to traditional treatment regimens (12).

The importance of minimizing glucocorticoid exposure cannot be overstated. Multiple studies have demonstrated that long-term, high-dose cumulative glucocorticoid use increases cardiovascular risk and organ damage in SLE patients (31–34), with cardiovascular events being major contributors to long-term adverse outcomes in LN. Therefore, the glucocorticoid-sparing effect observed with telitacicept could potentially translate to reduced long-term morbidity, improved quality of life, and enhanced treatment adherence.

In terms of safety, our study reported 13 adverse events, including three cases of pneumonia requiring hospitalization, all of which were reversible with appropriate intervention. No serious adverse events or severe infections occurred, suggesting that telitacicept combined with standard therapy does not significantly increase adverse events and has a favorable safety profile.

Our study has several limitations that should be considered when interpreting the results. First, as a single-center retrospective study with a relatively small sample size, selection bias may have influenced our findings, and the statistical power to detect differences between subgroups was limited. Second, the follow-up period was relatively short, particularly for the 52-week analyses which included only 16 patients; longer-term studies are needed to assess durability of response and long-term safety. Third, the lack of a control group receiving standard therapy without telitacicept limits definitive conclusions about the added benefit of telitacicept. Fourth, the progressive reduction in patient numbers during follow-up introduces potential attrition bias, as patients with better responses may have been more likely to continue treatment. Multicenter, prospective studies with larger sample sizes and longer follow-up periods are needed to confirm our findings.

In conclusion, this real-world study demonstrates that telitacicept combined with standard therapy for lupus nephritis shows promising efficacy and safety. The treatment was associated with high rates of complete and partial renal responses, significant reduction in proteinuria, decreased overall disease activity, substantial glucocorticoid-sparing effects, and improvement in immunological parameters. The therapy was well-tolerated with no serious adverse events, suggesting a favorable benefit-risk profile. Our findings suggest that telitacicept may represent an important addition to the therapeutic armamentarium for LN, particularly for patients with proliferative nephritis. Randomized controlled trials and larger, multicenter, long-term studies are warranted to further establish the role of telitacicept in the management of lupus nephritis and to identify patient subgroups most likely to benefit from this therapy.

## Data Availability

The raw data supporting the conclusions of this article will be made available by the authors, without undue reservation.
